# *Clostridium perfringens* sepsis after pancreatoduodenectomy: a case report

**DOI:** 10.1186/s40792-022-01402-z

**Published:** 2022-03-21

**Authors:** Goro Takahashi, Yoshiharu Nakamura, Tomohiro Hayakawa, Takashi Ono, Kazuhiko Endo, Hiroshi Yoshida

**Affiliations:** 1 Department of Surgery, Kamisu Saiseikai Hospital, 7-2-45 Shittechuo, Kamisu, Ibaraki 314-0112 Japan; 2 Department of Gastrointestinal and Hepato-Biliary-Pancreatic Surgery, Nippon Medical School, Tokyo, Japan

**Keywords:** *Clostridium perfringens*, Sepsis, Hepatic abscess, Intravascular hemolysis, Pancreatoduodenectomy

## Abstract

**Background:**

*Clostridium perfringens* sepsis associated with massive intravascular hemolysis has an extremely poor prognosis. We here report a case of *C. perfringens* sepsis associated with massive intravascular hemolysis that developed secondary to a post-pancreaticoduodenectomy (PD) hepatic abscess.

**Case presentation:**

A 70-year-old man with Type 2 diabetes underwent PD for an ampulla of Vater carcinoma. His postoperative course was uneventful. He was discharged on the 16th post-operative day (POD 16) after confirming no major abnormalities on abdominal contrast computed tomography (CT) on POD 14 or laboratory results on POD 16. Two days after discharge, he was readmitted because of fever and chills. Laboratory tests showed only a mild inflammatory reaction (white blood cell count, 11,980/mm^3^; C-reactive protein, 2.07 mg/dL). Abdominal CT showed an irregular, approximately 20-mm diameter, low-density area in the liver S6 region that had not been seen on a recent previous scan. We initially suspected postoperative cholangitis associated with biliary reconstruction and started empirical treatment with sulbactam/ampicillin after drawing blood for culture. Eight hours after admission, he developed septic shock with body temperature 40.0 ℃ and blood pressure 70/40 mm Hg. Laboratory findings showed a severe inflammatory reaction, severe anemia, and massive hemolysis (white blood cell count, 37,400/mm^3^; hemoglobin, 7.7 g/dL; total bilirubin, 8.05 mg/dL; direct bilirubin, 2.66 mg/dL; and lactate dehydrogenase, 1686 U/L). Hemoglobinuria was noted in the urinary catheter output. Repeat CT 9 h after admission showed the low-density area in S6 had become a gas-forming abscess. *C. perfringens* sepsis was strongly suspected on the basis of these findings and the abscess was drained percutaneously immediately after its diagnosis. His vital signs improved dramatically and he recovered within 24 h. Blood and abscess cultures grew *C. perfringens* 4 days after admission, leading to a definitive diagnosis of *C. perfringens* sepsis associated with massive intravascular hemolysis. He was discharged 18 days after admission. His sepsis has not recurred.

**Conclusions:**

*Clostridium perfringens* infection should be considered in patients who have undergone PD and present with gas-forming hepatic abscesses and/or sepsis associated with intravascular hemolysis. Prompt aggressive treatment is crucial, because *C. perfringens* infections can cause death within hours.

## Background

Clostridium perfringens sepsis has an extremely poor prognosis [1, 2]. Mortality reportedly ranges from 70 to 100% and is particularly high in patients with massive intravascular hemolysis [1]. We here report a patient who developed *C. perfringens* sepsis associated with massive intravascular hemolysis after pancreaticoduodenectomy (PD) who was successfully treated with prompt initiation of intravenous antibiotics, immunoglobulin infusion, and percutaneous abscess drainage.

## Case presentation

**Fig. 1:**
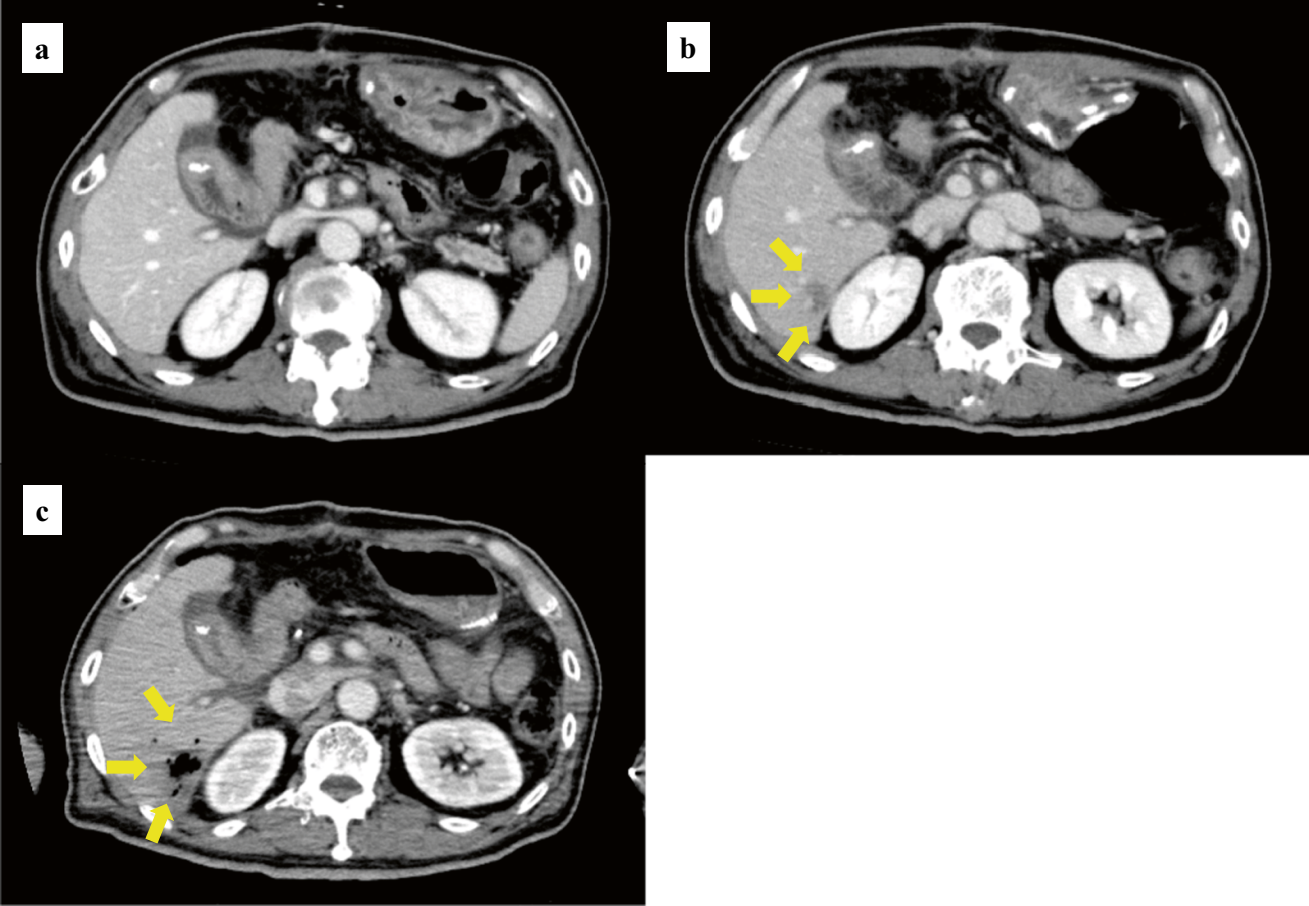
Computed tomography findings over time. **a** Postcontrast image showing no abnormal findings on POD 14, 2 days before discharge. **b** Image obtained when the patient was readmitted on POD18 showing an irregular, 20 mm, low-density area in the S6 region of the liver. **c** Image obtained 8 h after admission showing that the affected area has transformed into a gas-producing abscess

A 70-year-old man with Type 2 diabetes underwent PD for an ampulla of Vater carcinoma. The operation time was 345 min and blood loss volume 120 mL. His postoperative course was uneventful and no postoperative complications such as bile leakage and pancreatic fistula were detected. He was discharged on the 16th post-operative day (POD 16) without any extracorporeal drainage tube after confirming that there were no major abnormalities on abdominal contrast computed tomography (CT) on POD14 or laboratory results on POD16 (Table 1). The tumor was stage pT3a N0 M0 (stage IIa) according to the Union for International Cancer Control classification, 8th edition. Two days after discharge on POD18, he was readmitted because of sudden onset of high-grade fever and chills. During those 48 h, he had stayed at home, had no gastrointestinal symptoms, and had not eaten stale food. Although he appeared slightly confused at the time of examination, his vital signs were stable. His body temperature was 39.0 ℃, pulse rate 100 beats/min and regular, blood pressure 120/80 mmHg, and respiratory rate 15 breaths per minute with an oxygen saturation of 98% on room air. Abdominal examination was unremarkable. Laboratory tests showed a mild inflammatory reaction and liver dysfunction (Table 1). Abdominal contrast-enhanced CT showed an irregular, 20-mm diameter, low-density area in the liver S6 region, which had not been present on imaging performed 4 days previously (Fig. 1a, b). Post-PD cholangitis was suspected and empiric sulbactam/ampicillin (12.0 g daily) initiated after two sets of blood cultures had been drawn.

**Table 1:** Laboratory results at first discharge, readmission and 8 h after readmission

	First discharge POD 16	Readmission POD 18	8 h after readmission	Normal range
WBC (/mm^3^)	9120	11,980	37,400	3900 to 9800
RBC (× 10^4^/mm^3^)	401	391	242	410 to 530
Hb (g/dL)	11.8	11.7	7.7	14.0 to 18.0
Ht (%)	35.8	35.0	21.3	39 to 52
Plt (× 10^4^/mm^3^)	29.4	27.6	24.2	13 to 36
AST (IU/L)	31	44	405	8 to 38
ALT (IU/L)	86	94	216	4 to 44
T-bil (mg/dL)	0.42	0.53	8.05	0.2 to 1.2
D-bil (mg/dL)	N/A	0.23	2.66	0 to 0.4
ALP (U/L)	79	101	88	38 to 113
LDH (U/L)	N/A	179	1686	125 to 220
UN (mg/dL)	15.5	17.8	32.0	7.8 to 20
Cre (mg/dL)	0.65	0.66	0.93	0.6 to 1.1
CRP (mg/dL)	3.11	2.07	2.81	< 0.30

*ALP* alkaline phosphatase, *ALT* alanine aminotransferase, *AST* aspartate aminotransferase, *bil* total bilirubin, *Cre* creatinine, *CRP* C-reactive protein, *D-bil* direct bilirubin, *Hb* hemoglobin, *Ht* hematocrit, *LDH* lactate dehydrogenase, *N/A* not available, *Plt* platelet count, *POD* postoperative day, *RBC* red blood cell count, *T-UN* urea nitrogen, *WBC* white blood cell count

**Fig. 2:**
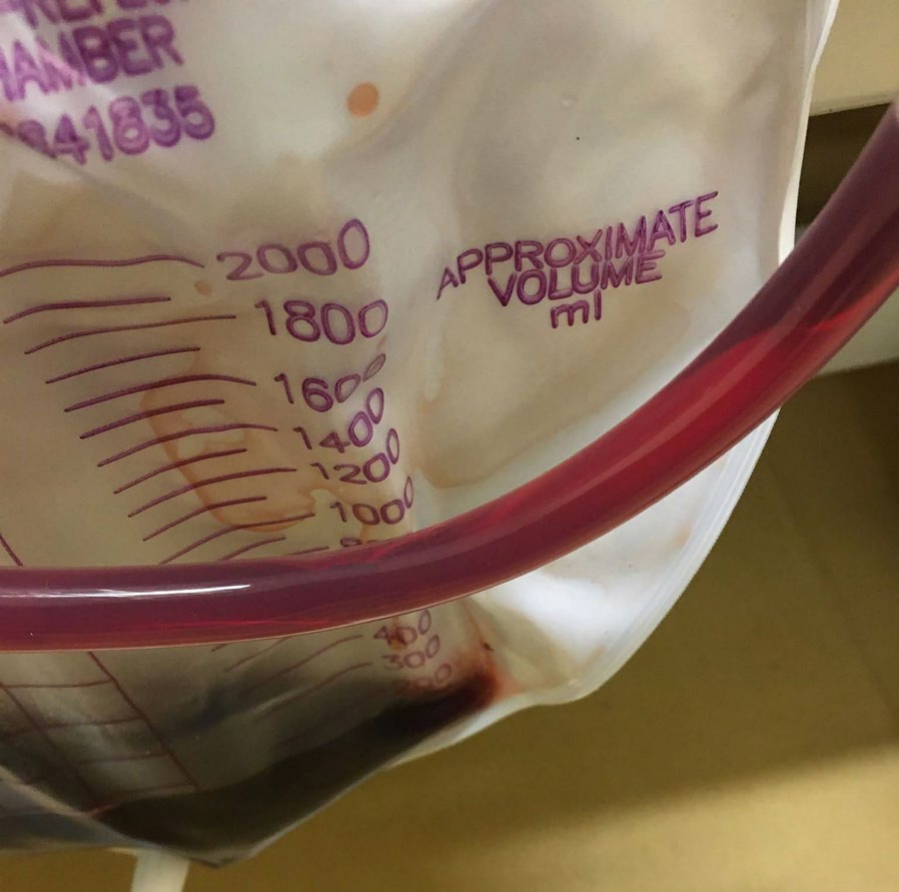
Hemoglobinuria associated with *Clostridium* *perfringens* sepsis-induced hemolysis

**Fig. 3:**
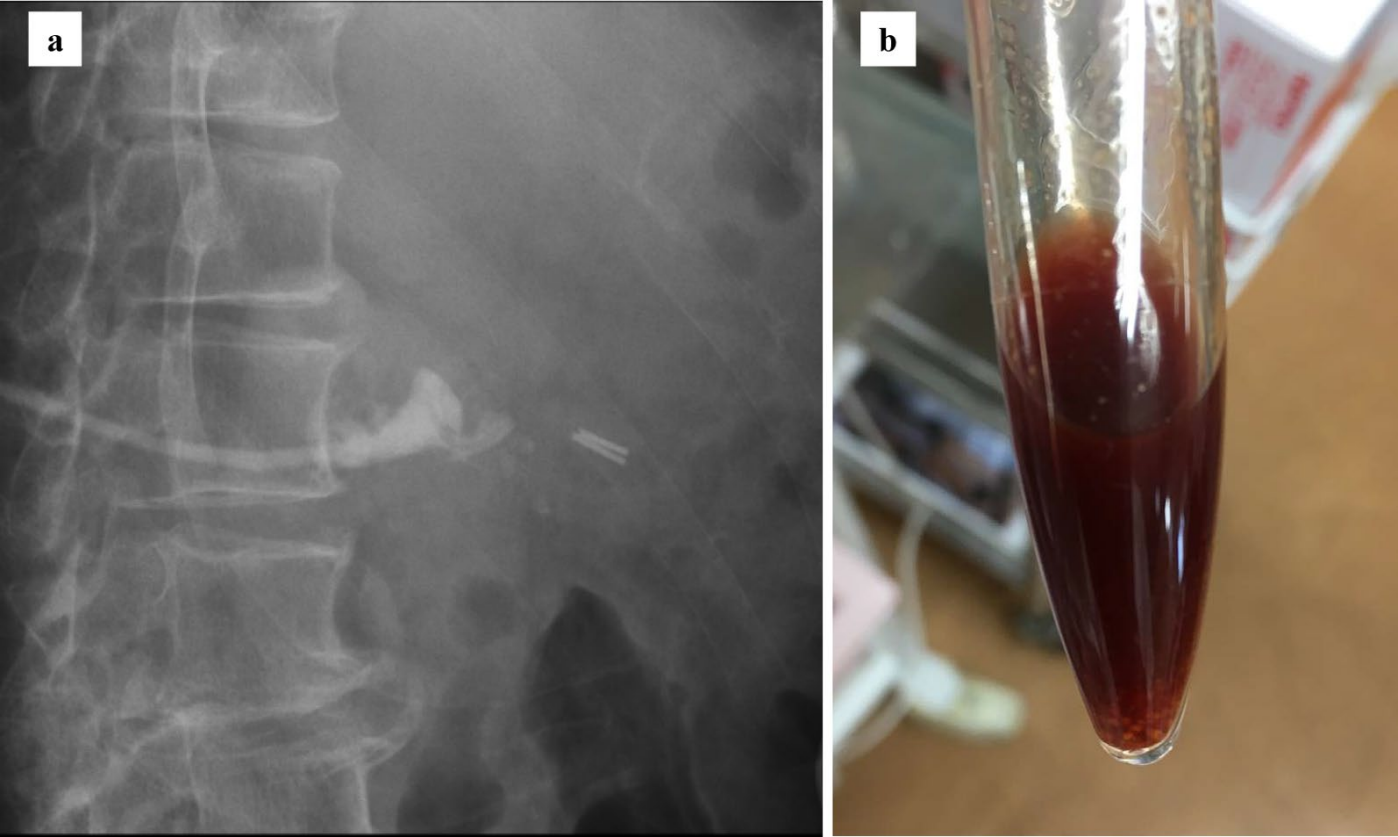
Percutaneous hepatic abscess drainage. **a** Percutaneous drainage was performed using an 8 Fr pigtail catheter. **b** Drainage fluid was red–black and had a foul odor

**Fig. 4:**
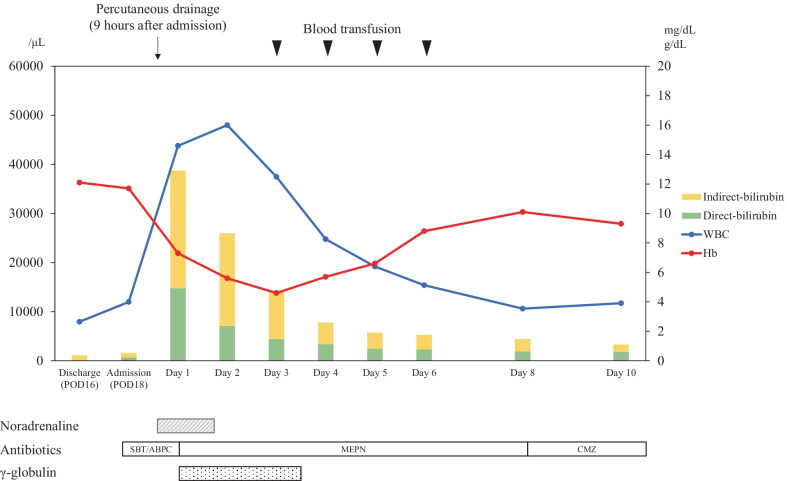
Clinical course. The blue line represents the white blood cell count (WBC) (left *y* axis) and the red line hemoglobin (Hb) concentrations (right *y*-axis). The bar graph represents serum bilirubin concentrations (right *y*-axis). After drainage of the hepatic abscess, the patient’s vital signs improved dramatically. Blood tests showed improvements in inflammation-related variables and a decrease in bilirubin concentration. *CMZ* cefmetazole, *MEPN* meropenem, *SBT/ABPC* sulbactam/ampicillin

Despite administration of these antibiotics, 8 h after admission the patient developed septic shock that required infusion of intravenous fluids and vasopressors. His body temperature was 40.0 ℃, blood pressure 70/40 mm Hg, heart rate 100 beats/min, and respiration rate 25/min. As shown in Table 1, laboratory findings were consistent with a severe inflammatory reaction, severe anemia, and massive hemolysis (white blood cell count, 37,400/mm^3^; hemoglobin, 7.7 g/dL; total bilirubin, 8.05 mg/dL; direct bilirubin, 2.66 mg/dL; and lactate dehydrogenase, 1686 U/L). Hemoglobinuria was noted in the urinary catheter output (Fig. 2). Direct and indirect Coombs testing for anti-red blood cell antibodies was negative. Nine hours after admission, follow-up enhanced CT showed transformation of the low-density area in the liver to a gas-forming hepatic abscess (Fig. 1c). *C. perfringens* sepsis being strongly suspected on the basis of these findings, meropenem (3.0 g daily) was substituted for the original antibiotics. We also administered intravenous immunoglobulin and drained the hepatic abscess percutaneously immediately after diagnosis, because this seemed to be the focus of infection. After initial drainage of about 5 mL of a dark red turbid liquid with a foul odor, there was little further drainage from the pig-tail catheter (Fig. 3). Twenty-four hours after drainage, the patient began to recover from his septic shock and blood tests showed improvement in inflammation-related variables and a decrease in bilirubin concentration (Fig. 4). Because his circulatory system function was maintained and to avoid the risk of α-toxin-related hemolysis, no blood was transfused until day 3. However, there were no adverse effects from transfusion. Blood and hepatic abscess cultures grew *C. perfringens* on day 4, leading to a definitive diagnosis of *C. perfringens* sepsis. The patient’s antibiotic regimen was changed to cefmetazole on day 8 on the basis of results of susceptibility testing. He was discharged on day 18 with some residual renal failure that resolved over the following 2 months. Drip infusion cholecysto-cholangiography (DIC-CT) 2 months after discharge showed no evidence of biliary anastomosis stenosis, such as dilation of the intrahepatic bile duct. Sepsis has not recurred in the 10 months since discharge.

## Discussion

Our patient illustrates two important clinical issues with respect to *C. perfringens* infection. First, *C. perfringens* should be suspected when treating a post-PD patient with a rapidly developing gas-producing abdominal abscess and/or sepsis with massive intravascular hemolysis. Second, prompt drainage is required for suspected *C. perfringens* abscesses.

Despite improvements in surgical techniques and perioperative care, the postoperative morbidity rate for PD remains at 40–50% [3–5]. Hepatic abscess is a relatively rare complication of PD, the reported frequency being 2.4–3.4% [5]. The pathogens most commonly responsible for post-PD hepatic abscesses are Enterococcus species, *Escherichia coli*, and *Klebsiella* *pneumoniae* [5]; there are few reports implicating *C. perfringens* [6]. *C. Perfringens*, an anaerobic gram-positive bacillus, is widely distributed in soil and sewage; however, it is also resident in the human digestive tract and genitourinary system. In addition, *C. perfringens* is reportedly present in the bile of up to 18% of patients undergoing biliary surgery [7]. It is known that these bacteria use extrinsic and intrinsic routes to cause infection. The former, which includes food poisoning and trauma, causes soft tissue infection (gas gangrene), whereas the latter causes non-traumatic sepsis as a result of bacterial translocation [8, 9]. *C. perfringens* sepsis rarely occurs in healthy individuals, being more likely to occur in patients with underlying disorders such as diabetes mellitus, malignancies, or immunosuppression, such as that related to chemotherapy [8, 9]. Therefore, in our patient, the hepatic abscess caused by *C. perfringens* may have been attributable to immunosuppression caused by surgical stress in addition to bacterial translocation from the intestinal tract, because there was no stenosis of the biliary anastomosis, which could be a cause of cholangitis, in the postoperative DIC-CT.

Early diagnosis of *C. perfringens* infection is difficult, because the symptoms are non-specific in the early phase. They include disturbed consciousness, epigastric pain, vomiting, and nausea [2]. In our patient, we initially diagnosed mild postoperative retrograde cholangitis associated with biliary reconstruction. In the future, greater availability of rapid polymerase chain reaction-based testing should assist with diagnosis [10]. Once sepsis has progressed in a patient with *C. perfringens* infection, α-toxin produced by these bacteria causes massive intravascular hemolysis, which can result in sudden death. Bunderen et al. reviewed 40 cases of *C. perfringens* sepsis with intravascular hemolysis reported after 1990, 32 (80%) of whom died, the median time to death being 8 h [1]. Our patient may have survived, because he was diagnosed rapidly and treated appropriately after development of sepsis. In addition, the focus of infection was relatively small.

Prompt drainage is vital for suspected *C. perfringens* abscesses. In a review of patients with *C. perfringens* septicemia, Kurasawa et al. [9] reported a 90% mortality in 30 patients with *C. perfringens* hepatic abscesses. Three of the six patients (50%) who underwent debridement or drainage survived. These authors advocated aggressive treatment of the infection focus when *C. perfringens* is suspected. Our patient’s abscess was in an accessible liver location (S6), enabling expeditious imaging-guided percutaneous drainage. After drainage of his abscess, his vital signs improved dramatically, enabling weaning off vasopressors within 24 h. Considering that mortality is so high and can occur within several hours [1, 9, 11–13], we believe that the focus of infection should be drained as quickly as possible, even in high-risk patients.

We consider that our patient’s rapidly progressive anemia was caused by α-toxin produced by *C. perfringens*. Although hemophagocytic syndrome associated with *C. perfringens* infection is a possible differential diagnosis [14], the presence of hemoglobinuria and indirect hyperbilirubinemia, and the absence of pancytopenia and hepatosplenomegaly suggest that his rapidly progressive anemia was caused by α-toxin produced by *C. perfringens*. Alpha-toxin, a zinc metalloenzyme composed of 370 amino acids, is the most important cause of hemolysis and gas gangrene among the six major *C. perfringens* toxin types [15, 16]. Alpha-toxin binds to host cell membranes in the presence of calcium ions [17] and directly hydrolyzes phosphatidylcholine and sphingomyelin proteins, which disrupts the cell membrane [18]. An additional effect of α-toxin is indirect activation of the mitogen-activated protein kinase kinase/extracellular-signal-regulated kinase pathway [18], which is involved in generating reactive oxygen species and tumor necrosis factor-α. These then activate the intrinsic pathway of apoptosis and promote inflammation [15, 19]. Although there is no established antimicrobial regimen for treating *C. perfringens* infection [20], clindamycin, metronidazole, and rifampicin are more effective than penicillin at reducing α-toxin release in mice [21]. In addition, erythromycin pretreatment reportedly reduced tumor necrosis factor-α release from activated neutrophils in a *C. perfringens* gas gangrene mouse model [22].

## Conclusions

*Clostridium perfringens* should be considered in post-PD patients with a rapidly developing, gas-producing, hepatic abscess and/or sepsis associated with massive intravascular hemolysis. *C. perfringens* infections progress rapidly and are usually fatal. Prompt and aggressive treatment is vital because death can occur within hours.

## Data Availability

All data analyzed in this study are included in this manuscript.

## Abbreviations

CT: Computed tomography
DIC-CT: Drip infusion cholecysto-cholangiography
PD: Pancreaticoduodenectomy
POD: Post-operative day
